# Knowledge, attitudes and practices (KAP) regarding leptospirosis among residents of riverside settlements of Santa Fe, Argentina

**DOI:** 10.1371/journal.pntd.0006470

**Published:** 2018-05-07

**Authors:** Tamara Ricardo, Laura C. Bergero, Esteban P. Bulgarella, M. Andrea Previtali

**Affiliations:** 1 Consejo Nacional de Investigaciones Científicas y Técnicas (CONICET), Santa Fe, Santa Fe, Argentina; 2 Departamento de Ciencias Naturales/ Facultad de Humanidades y Ciencias/ Universidad Nacional del Litoral, Santa Fe, Santa Fe, Argentina; 3 Observatorio Social/ Universidad Nacional del Litoral, Santa Fe, Santa Fe, Argentina; Universidad Nacional Mayor de San Marcos, PERU

## Abstract

**Background:**

Leptospirosis is a global and re-emerging zoonotic disease caused by *Leptospira* spirochetes that are shed into the environment by infected animals. Humans can get infected via contact with animal hosts or contaminated environment. In Argentina, the highest annual incidences were reported in the province of Santa Fe, where epidemic outbreaks occurred during flooding events. This study examined the knowledge, attitudes and practices (KAP) regarding leptospirosis among residents of riverside slum settlements from Santa Fe after a major flood.

**Methods and findings:**

A cross-sectional questionnaire was administered to 113 residents of 3 riverside settlements from Santa Fe. The influence of knowledge and attitudes regarding leptospirosis on the likelihood that an individual will use preventive practices were evaluated using linear mixed-effects models. The majority of respondents (83.2%) had previously heard about leptospirosis; however specific knowledge about leptospirosis was limited. The results of the modeling efforts, show that the likelihood of using preventive practices was associated with having greater knowledge score, but not with more positive attitudes. We also found that females were more likely to use safer practices than males.

**Conclusions:**

Even though the majority of respondents had heard about leptospirosis, a high percentage of them had limited knowledge regarding the severity of the disease and its prevalence in the region. Our results suggest that public health interventions in these riverside communities should focus on educating the public on the multiple dimensions of leptospirosis in order to attain greater adherence to preventive practices instead of intending to change the perceptions or attitudes towards the disease, which did not have a significant influence. The key challenge lies in identifying effective strategies to reach the high risk group for leptospirosis here that is male fishermen, who spend most of the time in precarious campsites on the river islands.

## Author summary

Leptospirosis is a zoonotic bacterial disease that has been recognized as a growing public health problem affecting mainly residents from slum settlements located in floodable areas. As such, it is considered a neglected disease that needs greater attention to reduce its global burden. A key step towards this purpose is to identify factors that influence the adherence to preventive practices regarding leptospirosis in endemic areas. We conducted a survey on residents of riverside settlements of the province of Santa Fe, an endemic area in Argentina, in order evaluate the knowledge, attitudes and practices regarding leptospirosis. Our results suggest that risky practices were performed mainly by men and that, contrary to our expectations, having a positive attitude towards leptospirosis does not appear to influence the likelihood of performing preventive practices, while greater knowledge about the disease does lead to safer practices. Public health officials should develop a comprehensive plan with diverse information, education and communication activities to promote a better understanding of the symptoms, treatment and prevention of leptospirosis by the various actors involved.

## Introduction

Leptospirosis is a zoonotic disease caused by *Leptospira* spirochetes. Pathogenic leptospires are excreted in the urine of mammalian hosts such as rodents, dogs and cattle and can persist in the environment for weeks or months [1–4]. Humans serve as incidental hosts, exposure may occur through direct contact with infected animal urine and tissues, or indirect contact with contaminated soil and water [1, 4, 5].

The contact with environmental sources of leptospires in urban and rural slum settlements can be increased by lack of basic sanitation, poor housing, crowding and extended time outdoors, together with heavy rainfall and flooding [6–12]. Furthermore, slum residents often engage in informal work such as small-scale construction, subsistence hunting or fishing, and food preparation for vending in the same areas where they reside [1, 2, 7, 10] or maintain subsistence livestock and chickens in their backyards [2, 4, 7, 8, 11], increasing the risk of environmental exposure.

Every year, between 500,000 and 1.03 million cases of leptospirosis are reported in the world, with a mortality rate over 10% [1, 13]. However, the global burden of leptospirosis is thought to be underestimated by several factors, including the broad clinical spectrum of the disease that mimics many other endemic infectious diseases such as dengue and malaria [1, 13–15]. Aditionally, many countries lack a case notification system or notification is not mandatory [1, 16, 17].

Latin America is one of the regions with the highest number of cases of leptospirosis in the world [14, 16], having reported 10,088 cases in 2014 of which 40.2% belonged to Brasil, followed by Perú, Colombia and Ecuador [16]. Even though Argentina reported 217 laboratory confirmed cases in 2014 [16], between 2005 and 2017, 14,319 suspected cases were reported to the National Health Surveillance System (SNVS) [18–23], being one of the leading countries in alerts of cases in Latin America [16].

The main risk factor for leptospirosis in Argentina is the persistent contact with flooded environments [8, 23–26]. Floodings may lead to disruption of health services and damage to households and water and sanitation networks, displacing populations and increasing the risk of exposure to rats and pathogens [2, 15].

The first case of leptospirosis in Argentina, was reported in the province of Santa Fe in 1915 [27]. Leptospirosis is considered an emerging public health problem in the country, and notification of suspected cases is mandatory [8, 23, 26]. The highest annual incidence rates of leptospirosis in Argentina occur in the province of Santa Fe [23, 26, 28], representing 46.4% of reported cases and 38% of confirmed cases in the country for the period 2012-2017 [18–22], with outbreaks registered following flooding events [23–26, 29, 30]. The number of severe cases of leptospirosis associated with pulmonary hemorrhage has increased in recent years [8, 26, 28].

Assessments of people’s knowledge of leptospirosis and health behavior provide critical information for disease prevention [9, 31–35]. In particular, surveys of knowledge, attitudes and practices (KAP) are useful public health tools to identify effective strategies for behavior change towards safer practices [32, 33, 36, 37]. Despite this, leptospirosis remains a neglected disease in Argentina and no studies have been conducted to assess the level of public awareness about the disease. The objectives of this study were to describe the knowledge, attitudes and practices regarding leptospirosis in riverside slum settlements from Santa Fe affected by a flood event and to evaluate the factors influencing preventive practices.

## Materials and methods

### Study location and population

The city of Santa Fe (31°38′0″S, 60°42′0″W) with a population of 391,231 in 2010 [38], is the capital of Santa Fe province, located in north-eastern Argentina in the junction of the Paraná and Salado rivers. Study sites comprised two riverside neighborhoods of Santa Fe and a settlement 30 km NE from the city. All three sites were located in the flood valley of the Paraná river, an area with high susceptibility to floods and different levels of deficiency in sanitary infrastructure.

**Fig 1:**
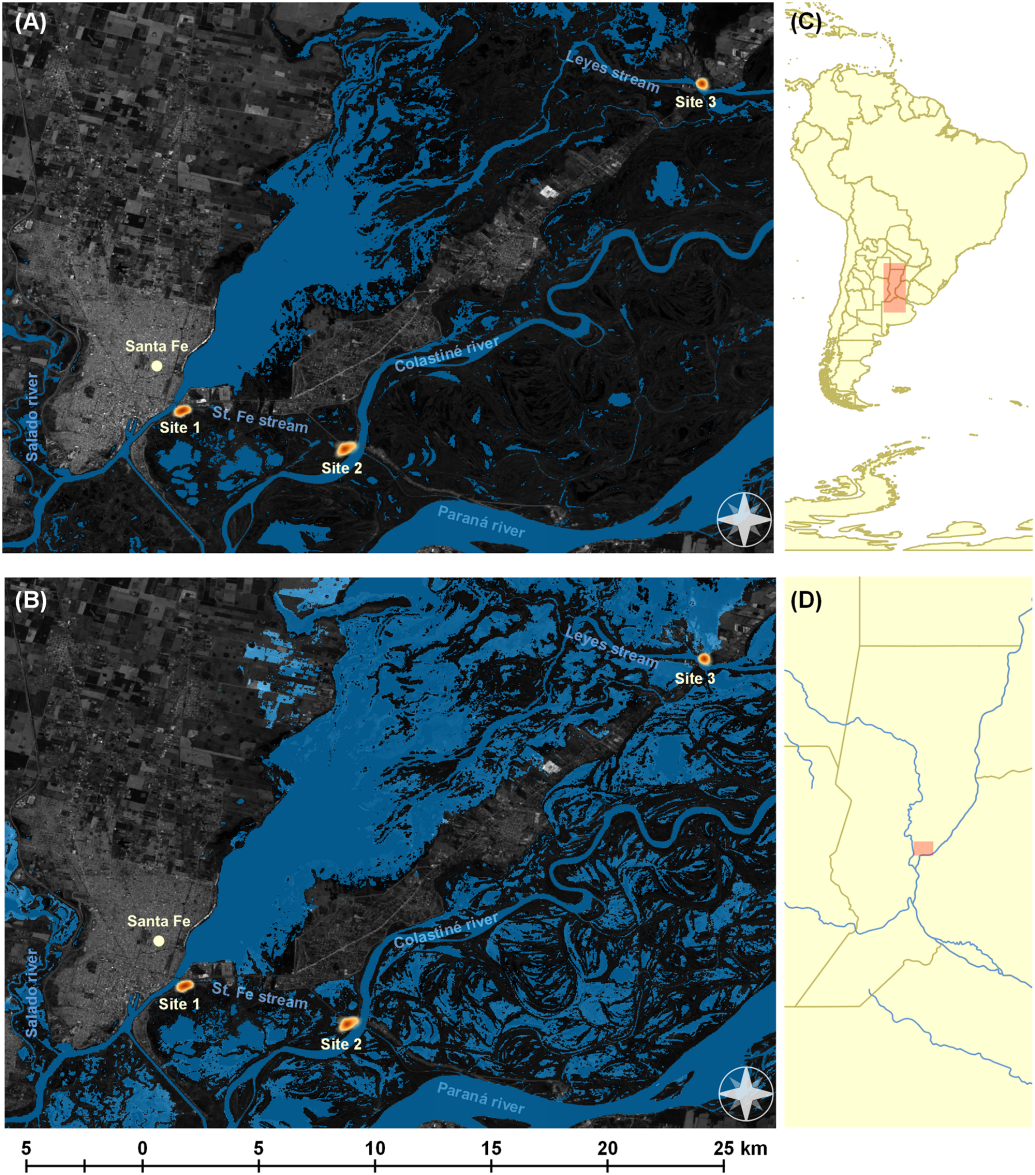
Flood map of the locations where the questionnaire was implemented. (A) Before the flood event; (B) During the flood event; (C) Location of the province of Santa Fe in Argentina; (D) Location of the study area in the province of Santa Fe. Accessible areas of study sites are overlaid as heat map where darker shades of orange indicate higher concentration of sampling units. Map generated with QGIS Geographic Information System. Satellite imagery was downloaded from Landsat8 OLI/TIRS downloaded from U.S. Geological Survey at: https://ers.cr.usgs.gov/. Vector layers were downloaded from Natural Earth at: http://www.naturalearthdata.com/.

A map was constructed to show how the landscape changed during the flood event (Fig 1) using QGIS 3.0 Girona [39] with the Semiautomatic Classification Plugin [40]. The base map and the raster layers of water bodies, before and during the flood event, were created from Landsat8 OLI/TIRS satellite imagery acquired from U.S. Geological Survey (https://ers.cr.usgs.gov). Vector layers were acquired from Natural Earth (http://www.naturalearthdata.com).

Site 1 corresponds to the neighborhood called La Vuelta del Paraguayo, located on the banks of the Santa Fe stream (Fig 1) with about 408 residents distributed in 64 households [41]. This site has water supply services and electricity but has no sewers, health centers, paved streets or public transportation [42]. Site 2 corresponds to Colastiné Sur, a riverside neighborhood located on the banks of the Colastiné river (Fig 1) with approximately 1018 residents distributed in 308 households [41]. This site has electricity, a health center, refuse recollection and public transportation but has no sewers, water supply services or paved streets [43]. Site 3 corresponds to a sector of the locality of Los Zapallos, this sector is located at the banks of the Leyes stream, 30 km NE of the city of Santa Fe (Fig 1). It consists of approximately 564 residents from 92 households. This site has electricity, water supply services, refuse recollection and a nearby health center (approximately at 1.5 km) but it does not have sewers or paved roads (Data obtained from the commune of Santa Rosa de Calchines).

### Data collection tools

**Fig 2:**
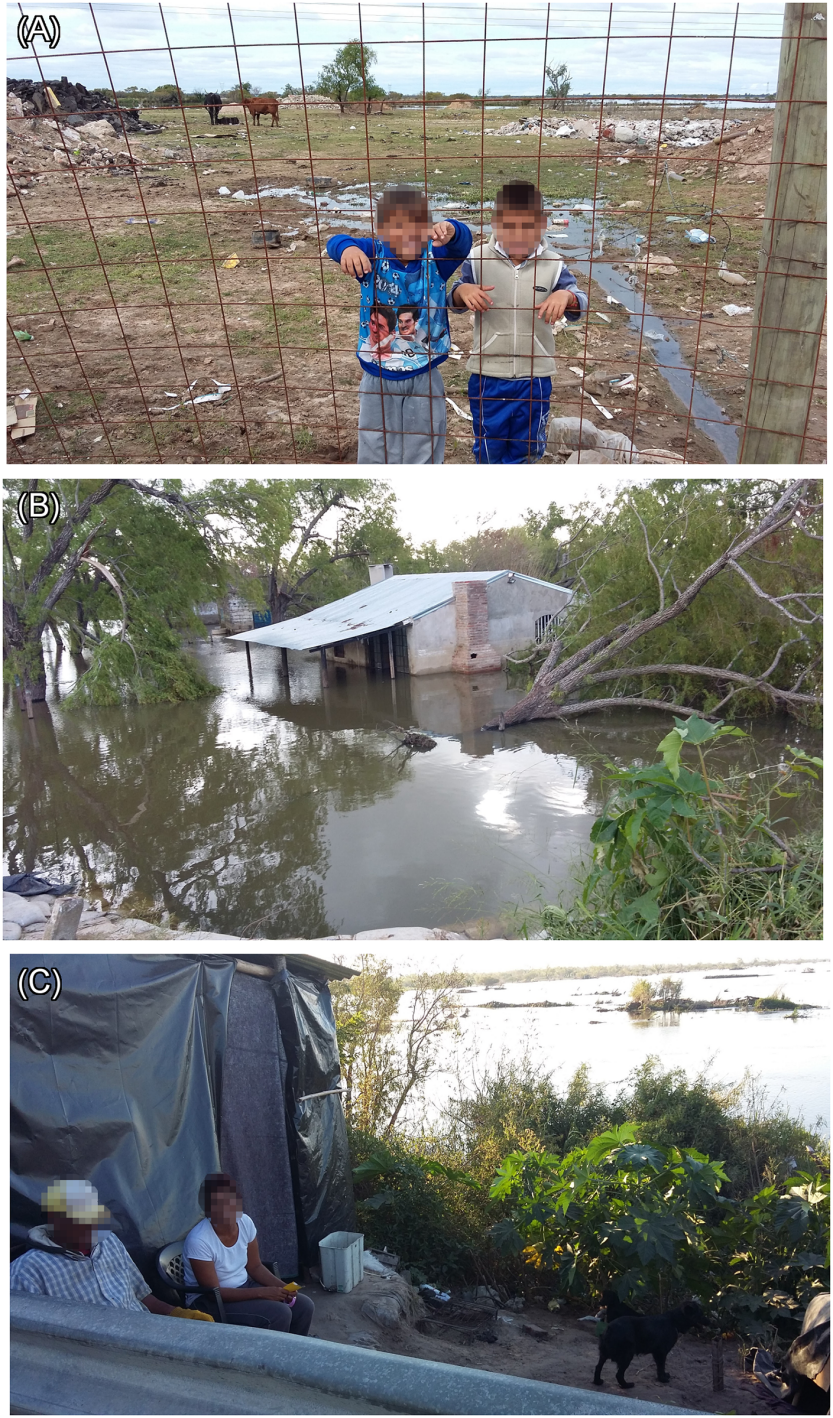
Study sites at the time of the questionnaire. (A) Children playing in a small dump-yard outside the evacuation center of Site 1; (B) Flooded household from Site 2; (C) Self-evacuated residents from Site 3.

Between March and May of 2016 we conducted a cross-sectional study assessing leptospirosis related KAP. Data was collected after a major flood event of the Paraná river that affected all study sites (Figs 1 and 2). Questionnaire participants were selected using a census sweep technique which allowed the sampling of both evacuated and non-evacuated households. Census sweeping was chosen as the sampling method to cover these small and clumped resident areas. We tried to minimize the potential influence of our presence on future questionnaire responses by conducting the census in the minimum number of days possible and following a particular direction for the sweeping design.

At each visit to the study sites, interviewer-administered questionnaires were used to gather necessary information from one resident per household. Similar to other published KAP studies, the questionnaire was conducted on residents who were at least 12 years old [9, 34, 35, 44, 45]. Residents were informed about the aspects of the research and a verbal consent was obtained from those willing to answer the questionnaire. Anonymity of the respondents and confidentiality of the data obtained were respected. Approval to conduct the survey was obtained from the local government units of Santa Fe and the Ethics committee of the Universidad Nacional del Litoral (CAID orientado 2013: “Socioecología de Leptospirosis”).

The questionnaire had been pre-tested in communities neighboring the study areas. The questionnaire consisted in 36 questions which included demographic factors such as age, sex, level of education attained, occupation and evacuation status. The questionnaire also included questions to asses the respondent’s KAP regarding leptospirosis (S1 Table). After the completion of the survey, an informative flier with most common symptoms of leptospirosis, modes of transmission and preventive actions was given and explained to each respondent (S1 Fig).

### KAP scores

Computation of practice scores was based on 8 items from the questionnaire (S1 Table: 2-7, 9, 11) ranging from a minimum of 0 points to a maximum of 14 points. A low score indicated risky behaviors or habits, while a high score was indicative of safer practices. Frequency of activities such as fishing, hunting and handling livestock, gathering firewood and gardening were categorized in: frequently (at least once a week), rarely (less than once a month) or never and were given a score from 0 to 2 respectively. Performing the above activities barefooted subtracted 2 points to the practice scores, while using footwear that is not water-proofed subtracted 1 point and using boots or waders did not subtract points. An extra point was added to the practice scores when the person indicated avoiding situations that are thought to increase transmission risk, such as going to the river islands, spending the night on the islands, walking barefooted through flood water or using river water for consumption or to clean or swim.

The computation of knowledge and attitude scores was restricted to those respondents who reported having heard about leptospirosis. Knowledge score increased as the person knew more about the disease. It was based on 7 items from the questionnaire (S1 Table: 14, 23-28) covering general aspects of leptospirosis, including knowledge on symptoms, transmission and preventive actions with a minimum of 0 and a maximum of 22 points. We included open-ended questions that allowed multiple answers and were scored as the sum of correct minus the incorrect answers. We also included closed-ended questions that were scored as 1 if the answer was “Yes” and 0 if the answer was “No/Does not know” being “Yes” the correct answer.

Attitude scores increased as the person responding the questionnaire communicated greater awareness of the risk and/or a greater tendency to act if symptoms appear or during an outbreak. Attitude scores were based on 7 items from the questionnaire (S1 Table: 16-17, 29-33) with a minimum of 0 and a maximum of 13 points, and included questions regarding perceptions of leptospirosis prevalence in the area and reactions to a potential leptospirosis outbreak that were scored from 0 to 2; questions regarding the perceived risk of leptospirosis in comparison to dengue, and the propensity of seeking medical attention in case of febrile symptoms were scored from 0 to 1. The crude scores for knowledge, attitudes, and practices were expressed as percentages dividing by the maximum score possible for each category and multiplying by 100.

### Data analyses

Data was entered using Microsoft Access, then cleaned and analyzed with R software version 3.4.1 [46]. Results were presented as frequency (%) for categorical variables and as mean ± standard deviation (SD) or median (IQR) for KAP scores. To compare KAP scores between sites we used analysis of variance (ANOVA) followed by Tukey’s post comparison tests or Kruskal-Wallis ANOVA followed by Dunn’s posts comparison tests to asses if there were differences between sites. The level of statistical significance was set at P≤0.05.

The factors associated with practice scores were evaluated using linear mixed-effects models (LMM) with site as random intercept, using the *lme4* package [47]. Respondents who reported hearing about leptospirosis and had no missing data on socio-demographic characteristics were included in the analysis. A list of candidate models was obtained using a manual step-backward procedure from a full model based on the Kenward-Roger approach, using the *pbkrtest* package [48]. The full model included the respondent’s sex, age, education attained, a dummy variable coded as 1 if the respondent worked outside of his/her home and 0 if not, a dummy variable coded as 1 if the respondent used media (television, radio, internet, newspaper) as source of health information or 0 if not, a dummy variable coded as 1 if the respondent used health services as source of health information and 0 if not, a dummy variable coded as 1 if the respondent knew someone that had leptospirosis and 0 if not, plus the respondent’s knowledge score and attitudes score. Models were then compared using second-order Akaike Information Criteria (AICc) [49] with the *MuMIn* package [50]. Inferences were derived from the most parsimonious model among the candidate models with a ΔAICc <4, and was refitted by Restricted Maximum Likelihood (REML) in order to obtain coefficient estimates for the random and fixed effects parameters.

## Results

### Socio-demographic characteristics

A total of 113 persons from the three study sites responded the survey, representing both evacuated (62.8%) and non-evacuated (37.2%) households (Table 1). The majority of the respondents (61.1%) were female and had primary school as the highest level of education attained (65.5%; Table 1). The ages of the respondents ranged from 12 to 77 years old with a median of 37 years old (IQR: 27-52).

**Table 1:** Frequencies (%) of socio-demographic characteristics and evacuation status of the respondents (n = 113).

Variable	Site 1 n = 26	Site 2 n = 46	Site 3 n = 41	Overall n = 113
**Sex**				
Female	15 (57.7)	27 (58.7)	27 (65.9)	69 (61.1)
Male	11 (42.3)	19 (41.3)	14 (34.1)	44 (38.9)
**Age group (years)**				
<18	1 (3.8)	5 (10.9)	5 (12.5)	11 (9.8)
18-29	3 (11.5)	9 (19.6)	8 (20.0)	20 (17.9)
30-44	10 (38.5)	15 (32.6)	11 (27.5)	36 (32.1)
45-64	8 (30.8)	13 (28.3)	13 (32.5)	34 (30.4)
≥65	4 (15.4)	4 (8.7)	3 (7.5)	11 (9.8)
**Education attained**				
Illiterate/Incomplete primary school	5 (19.2)	5 (10.9)	11 (26.8)	21 (18.6)
Primary school	18 (69.2)	31 (67.4)	25 (61.0)	74 (65.5)
High school	3 (11.5)	10 (21.7)	3 (7.3)	16 (14.2)
Data Missing	0 (0.0)	0 (0.0)	2 (4.9)	2 (1.8)
**Occupation**				
Employed	6 (23.1)	12 (26.1)	5 (12.2)	23 (20.4)
Builder	1 (3.8)	4 (8.7)	1 (2.4)	6 (5.3)
Fisherman	3 (11.5)	4 (8.7)	11 (26.8)	18 (15.9)
Student	1 (3.8)	5 (10.9)	7 (17.1)	13 (11.5)
Housewife/Unemployed/Retired	15 (57.7)	21 (45.7)	15 (36.6)	51 (45.1)
Data Missing	0 (0.0)	0 (0.0)	2 (4.9)	2 (1.8)
**Evacuee**				
No	1 (3.8)	22 (47.8)	19 (46.3)	42 (37.2)
Yes	25 (96.2)	24 (52.2)	22 (53.7)	71 (62.8)

Housewives, unemployed, retired and students represented 58.4% of the respondents. Of the respondents who worked outside of their home, 15.9% were subsistence fishermen, 5.3% worked in small-scale construction and 20.4% were engaged in other activities such as working at small retail business, domestic services and municipal employment (Table 1).

### Knowledge, attitudes and practices

**Fig 3:**
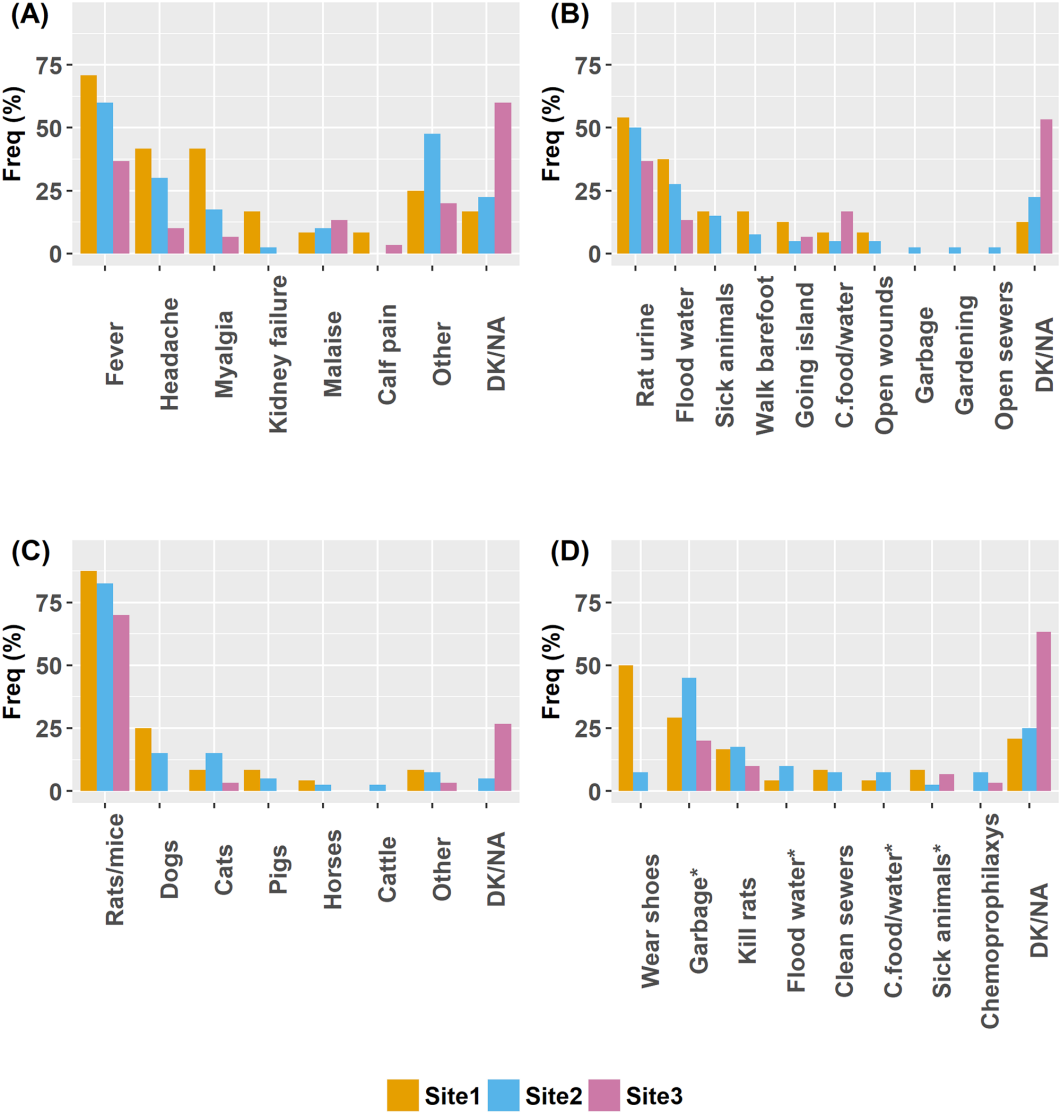
Knowledge about leptospirosis among respondents from three riverside settlements (n = 94). (A) Leptospirosis symptoms reported; (B) Environmental modes of transmission identified; (C) Suspected animal hosts mentioned; (D) Preventive measures mentioned. C.: Contaminated; *Avoidance of.

Ninety four (83.2%) respondents reported having previously heard of leptospirosis, and almost half of them (47.9%) knew at least one person who had the disease. The majority of respondents who had heard about leptospirosis identified it as a disease associated with rats (71.3%) and were aware that leptospirosis has a cure (72.3%) but it can be fatal (80.9%). The symptoms of leptospirosis that were frequently identified included fever (55.3%) and headache (26.6%) (Fig 3A). Almost a third of the respondents (29.8%) were not able to describe a mode of transmission. Of those who responded the question about transmission, the majority identified rats and mice as the main animal hosts (79.8%) and the urine of these animals as the main mode of transmission (46.8%) (Fig 3B and 3C). When asked about preventive actions, 36.2% of the respondents were unable to mention a preventive action and only 5.3% mentioned avoiding contact with flood water (Fig 3D). Overall mean knowledge score was 33.9% (SD±15.9%), ANOVA test yielded significant differences among site (P<0.001), with Site 1 (42.6%, SD ±14.6) and Site 2 (37.5%, ±13.3%) having significantly higher scores than Site 3 (22.3%, ±13.5%).

When asked about where they have heard about leptospirosis, almost half of the respondents (48.9%) reported using the media (television, radio, newspapers, internet) as their source of information, followed by health services (36.2%), relatives and neighbors (29.8%) and schools (14.9%).

Regarding the attitudes about leptospirosis, 52 (55.3%) respondents believed that there are few cases per year but 57 (60.6%) assumed that there could be an epidemic outbreak (Table 2). When asked how they would act in the face of a possible outbreak, 53 (56.4%) respondents said they would be afraid of becoming infected and 80 (85.1%) said they would be able to take preventive measures. The majority of respondents considered that dengue is more prevalent than leptospirosis in the area (59.6%), yet there was not a notable distinction on how they perceived their risk to these diseases, approximately a third of the respondents felt more at risk of leptospirosis, a third more at risk of contracting dengue, and a third felt equally at risk to both diseases (Table 2). While 55.3% of the respondents considered dengue to be a more severe disease than leptospirosis, 17% of the respondents considered them equally severe (Table 2). Of the 94 respondents, 16% were not able to respond the question regarding how prevalent they think these diseases are in the area, 12.8% were not able to respond to which of the two they felt more at risk and 7.4% were not able to respond the question about the severity of leptospirosis and dengue (Table 2). On the other hand, we found that the majority of the respondents usually seek medical care (77%), and when asked if they would seek it in case of persistent fever 96.4% gave an affirmative answer. Overall median attitudes score was 76.9% (IQR 30.8-100%) and Kruskal-Wallis ANOVA yielded no significant differences between sites (P = 0.26).

**Table 2:** Frequencies (%) of attitudes towards leptospirosis among respondents that heard about leptospirosis (n = 94).

Variable	Site 1 n = 24	Site 2 n = 40	Site 3 n = 30	Overall n = 94
**How many cases are reported yearly?**				
Large number of cases	9 (37.5)	13 (32.5)	2 (6.7)	24 (25.5)
Few cases	9 (37.5)	22 (55.0)	21 (70.0)	52 (55.3)
Do not know	6 (25.0)	5 (12.5)	7 (23.3)	18 (19.1)
**Could there be an outbreak?**				
Yes	21 (87.5)	22 (55.0)	14 (46.7)	57 (60.6)
No	2 (8.3)	11 (27.5)	7 (23.3)	20 (21.3)
Not sure	1 (4.2)	7 (17.5)	9 (30.0)	17 (18.1)
**Which is more prevalent?**				
Leptospirosis	5 (20.8)	6 (15.0)	2 (6.7)	13 (13.8)
Dengue	8 (33.3)	27 (67.5)	21 (70.0)	56 (59.6)
Same	4 (16.7)	3 (7.5)	3 (10.0)	10 (10.6)
Do not know	7 (29.2)	4 (10.0)	4 (13.3)	15 (16.0)
**Which are you more exposed to?**				
Leptospirosis	8 (33.3)	12 (30.0)	8 (26.7)	28 (29.8)
Dengue	10 (41.7)	13 (32.5)	7 (23.3)	30 (31.9)
Same	5 (20.8)	12 (30.0)	7 (23.3)	24 (25.5)
Do not know	1 (4.2)	3 (7.5)	8 (26.7)	12 (12.8)
**Which is a more severe disease?**				
Leptospirosis	6 (25.0)	7 (17.5)	6 (20.0)	19 (20.2)
Dengue	13 (54.2)	23 (57.5)	16 (53.3)	52 (55.3)
Same	4 (16.7)	7 (17.5)	5 (16.7)	16 (17.0)
Do not know	1 (4.2)	3 (7.5)	3 (10.0)	7 (7.4)

**Fig 4:**
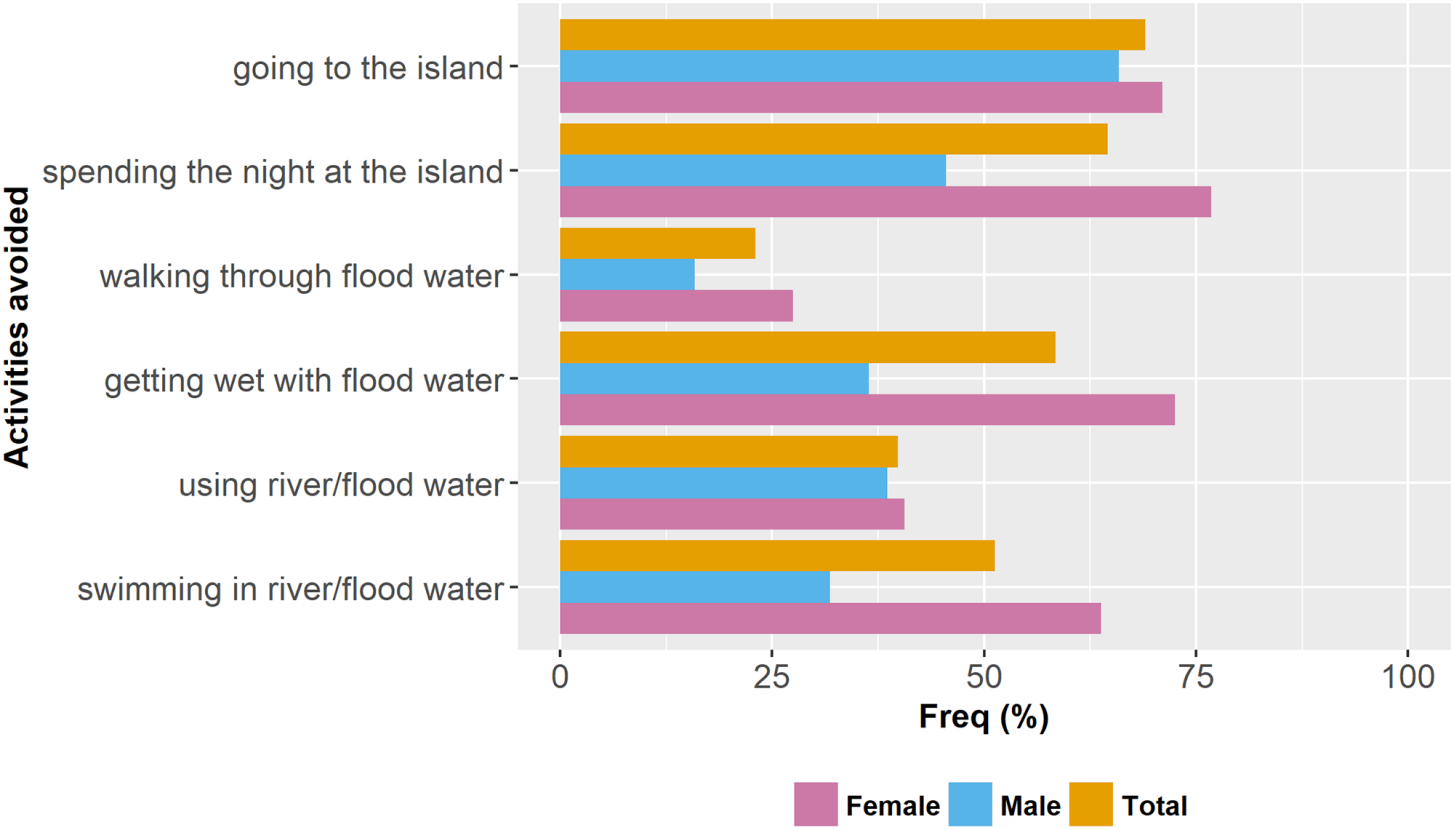
Avoidance of risky practices among male and female respondents (n = 113).

For preventive practices, 54 (48.7%) out of 113 respondents reported never going fishing, 94 (83.2%) reported never going hunting or handling livestock, 61 (64%) reported never doing gardening and 62 (54.9%) reported never collecting firewood. Differences between genders were observed in the frequencies of fishing and hunting (S2 Table). Of those respondents that reported performing one or more of those activities (n = 85), 44 (51.8%) wore inappropriate footwear, 36 (42.4%) wore boots or wading suits and 5 (5.9%) went barefooted. With regard of avoidance of risk practices, 58 (51.3%) respondents reported not going to the river islands, 73 (64.6%) reported not spending the night at the island, 26 (23%) reported avoiding to walk through flood water, 45 (39.8%) reported avoiding to get their feet wet on flood water, 78 (69%) reported to avoid the use of water from the river or flood water to drink or clean and 66 (58.4%) avoided to swim in the river or flood water (Fig 4). Differences between genders were observed in the avoidance of some risk situations (Fig 4; S2 Table). Overall median practices score was 57.1% (IQR 0-100%) and Kruskal-Wallis ANOVA yielded no significant differences between sites (P = 0.08).

### Factors affecting leptospirosis preventive practices

The adherence to leptospirosis preventive practices was assessed by modeling practices score as a function of 9 independent variables: sex, age, education and occupational status of the respondent, whether the respondent uses media as source of leptospirosis information, whether the respondent uses health services as source of leptospirosis information, whether the respondent knows someone that has had leptospirosis, knowledge score and attitudes score. Manual step-backward procedure yielded a list of 10 models, from which the most parsimonious model was selected (Table 3).

**Table 3:** Candidate linear mixed-effects models to explain variability on practices score using site as random intercept (n = 92).

Model	K	AICc	Δi	Wi	logLik
*sex + health.center + knowledge*	6	837.50	0.00	0.35	-412.26
*sex + knowledge*	5	838.12	0.62	0.26	-413.71
*sex + media + health.center + knowledge*	7	838.60	1.10	0.20	-411.63
*sex + employed + media + health.center + knowledge*	8	839.77	2.26	0.11	-411.02
*sex + employed + media + knowsSO + knowledge*	9	841.34	3.83	0.05	-410.57
*sex*	4	844.30	6.79	0.01	-417.92
*sex + education + employed + media + health.center + knowsSO + knowledge*	11	844.79	7.28	0.01	-409.74
*sex + education + employed + media + health.center + knowsSO + knowledge + attitudes*	12	846.89	9.39	0.00	-409.47
*sex + age + education + employed + media + health.center + knowsSO + knowledge + attitudes*	13	849.16	11.66	0.00	-409.25
*null*	3	865.21	27.70	0.00	-429.47

**K**: Number of effective parameters; **AICc**: Akaike’s bias-adjusted information criteria;**Δi**: differences in AICc between the candidate model and the best model; **Wi**: Akaike weights; **logLik**: log-Likelihood.

*KnowsSO*: whether the respondent knows someone that had leptospirosis

In the final model, practices score was negatively associated with male sex and positively associated with knowledge score (Table 4). In this model, fixed effects alone explained 28.43% of the variation while both fixed and random effects explained 29.87% of the variation (Table 4), residuals were normally distributed with a mild outlier.

**Table 4:** Parameter estimations for the coefficients of the most parsimonious model for practices score (n = 92).

Estimate	Coefficient (95% CI)
Intercept	46.48* (34.30; 58.67)
Sex: Male	**−** 25.50* (−34.66; −16.35)
Knowledge score	0.47* (0.17; 0.77)
AIC	830.84
logLik	-410.42
Nom. obs	92
Num. groups	3
Var. Site (Intercept)	9.88
Var. Residual	481.67
R^2^(m)	28.43%
R^2^(c)	29.87%

*p < 0.05; R^2^(m): marginal R-squared; R^2^(c): conditional R-squared.

## Discussion

To our knowledge, this is the first study that aimed at describing the knowledge, attitudes and preventive practices associated with leptospirosis in Argentina. Our study, conducted on riverside settlements of the province of Santa Fe, provides relevant information on the risk of leptospirosis among communities from an endemic area highly vulnerable to floods [23–25, 28–30]. The majority of the respondents in this study had heard about leptospirosis (83.2%), however, many of them were not able to describe leptospirosis symptoms (33%), nor modes of transmission (29.8%) or preventive actions (36.2%). In order for people in these communities to be better prepared to avoid infection or disease progression it is critical to increase the knowledge on these key aspects as previously suggested in studies from Chile [51], Philippines [32], Trinidad [35], Malaysia [33, 37] and Sri Lanka [44].

In terms of recognizing leptospirosis risk factors, only 25.5% of the respondents mentioned contact with flood water as a mode of transmission. Given that this is considered the main mode of transmission in the region [23–26], it is concerning to find that only a small proportion of the population is aware of this risk. These communities do not have paved roads and most of them have open sewers, commonly known as “zanjas”, that contain stagnant water most of the time. After large rainfall events, dirt roads are filled with puddles and rainfall is mixed with water from the open sewers and/or ponds. In the three study communities it is a common practice to cross these puddles barefooted (60.2%) which increases transmission risk.

Many respondents work in the informal sector, mainly in outdoor activities such as fishing, hunting and gardening, half of them did not use adequate footwear. Other studies have found that protective gear is often not used due to difficulty in acquiring it or because people feel that it is uncomfortable [9, 34, 44]. In our study communities, the use of adequate footwear may increase with a greater awareness that leptospirosis can be acquired through contact with contaminated water.

In this region, leptospirosis is also called “the disease of the rats” as most people consider that rats play an important role in disease transmission. In our study, 79.8% of the respondents mentioned rats as the source of leptospirosis to humans. In agreement with other studies [33, 44, 52], only a low percentage of respondents recognized dogs (12.8%) and cattle/pigs (5.4%) as animal sources of leptospirosis. This is of special concern considering that most households have several domestic animals, which typically includes several dogs and some individuals of livestock raised in the vicinity of the household. Additionally, *L. interrogans* serovar Canicola was isolated from cases of severe to lethal leptospirosis in the area [28, 53]. Furthermore, only a small percentage of the dog population on these communities is vaccinated against leptospirosis [54, 55].

Most respondents stated that only a few cases of leptospirosis occur yearly in the province of Santa Fe but believed that it was possible for an epidemic outbreak to occur in the area. This perception is in agreement with the data published by the National Health Surveillance System (SIVILA), that shows a total of 27 confirmed cases of leptospirosis in the province of Santa Fe between the epidemiological weeks 1 and 20 of 2016, of which 12 belonged to the department La Capital, where the study area is located [56–58]. However, if we consider the diversity of clinical manifestations of this disease and the limitations of laboratory diagnosis in the area, it is probable that leptospirosis is under-reported here [14, 28, 53].

Similarities between dengue and leptospirosis symptoms and environmental settings are likely contributing to the underestimation of the number of leptospirosis cases [33, 35, 59–61]. Dengue outbreaks during floods such as the 2016 flooding event of the Paraná river [56], may lead to leptospirosis cases being misdiagnosed as dengue. Between the epidemiological weeks 1 and 20 of 2016, 1324 dengue cases were reported for the province of Santa Fe [56]. We conducted the questionnaire during this dengue fever outbreak, when a massive campaign was in place to warn the public about the risk of dengue fever. In this scenario, is was surprising to find that approximately 30% of the respondents considered that they were are a greater risk of contracting leptospirosis and 25.5% considered that they were equally exposed to both pathogens. Finally, another factor that may contribute to the underestimation of the number of leptospirosis cases is the fact that the environmental setting of these riverside communities restricts the accessibility to health care services and laboratory diagnosis.

Overall, knowledge about leptospirosis appeared to decrease as the distance to the city of Santa Fe increases. This could be attributed to a greater distance to the hospitals and to a more limited access to information by the inhabitants of rural areas [62–64], even though all of them are settlements with apparently similar living conditions. Site differences were not observed in people’s attitudes and practices.

Our modeling efforts for explaining the variation in using preventive practices suggest that men have higher risk of contracting leptospirosis than females. In contrast to males, most of the female surveyed said they never went fishing (63.8%), never went hunting (94.2%), never go to the island (63.8%) or spent the night at the island (76.8%) and do not swim in the river (72.5%) (S2 Table). While in other activities such as collecting firewood, gardening and crossing puddles, no significant differences were observed between men and women (S2 Table). We also found that many of the men are engaged in fishing (31.8%) and most of the women are either housewives or unemployed (63.8%). This is in agreement with other studies that found a greater probability of infection in men than in females and have provided as a possible explanation to this gender differences the greater engagement of males in outdoor recreational and/or labor activities [1, 7, 11, 14, 25, 28, 32, 33, 44]. We also found that the likelihood of using preventive practices increased with knowledge. Most studies on leptospirosis KAP were descriptive and did not tried to identify factors that may influence the predisposition to use preventive practices [9, 33, 35, 37, 44, 65]. Our results are consistent with those of Arbiol et al. [32], Lau et al. [7] and other studies regarding zoonotic disease prevention practices that show that greater knowledge about the disease results in a greater adoption of preventive practices [31, 45].

The findings should be considered within the limitations of the study. The relatively homogeneous socio-demographic composition of the sample may have impacted on the significance of socio-demographic parameters on preventive practices. Another limitation of this study was the small sample size that may have precluded us from detecting a meaningful effect of other socio-demographic factors. In regards to generalization, our conclusions can only be extrapolated to similar riverside settlements in the region, where subsistence fishing is a common occupation among residents.

The results of this study suggest that increasing knowledge about leptospirosis is key for promoting desired, positive behaviors in the community, rather than changing the attitudes towards the disease. Thus, our study underscores the importance of implementing a diverse array of information, education and communication activities to achieve a better understanding of the symptoms, treatment and prevention of leptospirosis by the various actors involved. Health education should reach healthcare providers and the general public, in particular high-risk groups. In these riverside communities, a key challenge lies in identifying effective strategies to reach the principal high-risk group for leptospirosis: males who work or perform recreational activities outdoors and spend time in precarious campsites in the river islands. In this respect, one promising and innovative approach is the development and implementation of social marketing campaigns that use concepts and tools from marketing to reach the public and try to “promote socially beneficial behavior change” [66]. This strategy has been applied successfully to increase global awareness of the Chagas disease issues in a campaign that involved the participation of popular artists and athletes [67]. These campaigns should be designed for specific communities, given that the barriers to engaging in leptospirosis preventive practices are known to differ among communities [51]. For the present study, we developed a flier tailored to the riverside communities (see S1 Fig that was explained after conducting the questionnaire, and we offered outreach workshops for the local schools. These instances provided opportunities to exchange ideas about preventive practices that seemed more attainable.

To identify means to improve public access to information on prevention practices and to adequate health care for early diagnosis and treatment, it is necessary to conform teams composed by health workers, researchers and policy makers. The interdisciplinary approaches to public health issues, promote a better understanding of the problems and provide comprehensive solutions for different scenarios [68, 69]. In order for these teams to develop more effective public health policies and programs, they need access not only to high-quality epidemiological data [13], but also to relevant sociocultural information [70].

## Supporting information

S1 Table Questionnaire implemented at the study sites (in Spanish). The questions used in the construction of knowledge score are highlighted in yellow, those used in the construction of attitudes score are highlighted in green, and those used in the practices score are highlighted in pink.
(PDF)

S2 Table Frequencies (%) of risk practices performed and risk situations avoided by female and male respondents (n = 113). (PDF)

S1 Fig Informative flier given and explained to survey respondents (in Spanish). (PDF)

## References

[1] Haake DA, Levett PN. Leptospirosis in humans. In: Curr. Top. Microbiol. Immunol. vol. 387; 2015 p. 65–97. 25388133 10.1007/978-3-662-45059-8_5 PMC4442676

[2] Céspedes M. Leptospirosis: Enfermedad Zoonótica Reemergente. Rev Peru Med Exp Salud Publica. 2005;22(4):290–307.

[3] Trueba G, Zapata S, Madrid K, Cullen P, Haake D. Cell aggregation: A mechanism of pathogenic Leptospira to survive in fresh water. Int Microbiol. 2004;7(1):35–40. 15179605

[4] WHO. Human leptospirosis: guidance for diagnosis, surveillance and control. WHO Libr. 2003;45(5):1–109.

[5] Ko AI, Goarant C, Picardeau M. Leptospira: The dawn of the molecular genetics era for an emerging zoonotic pathogen. Nat Rev Microbiol. 2009;7(10):736–747. doi: 10.1038/nrmicro2208 19756012 10.1038/nrmicro2208 PMC3384523

[6] Leibler JH, Zakhour CM, Gadhoke P, Gaeta JM. Zoonotic and Vector-Borne Infections Among Urban Homeless and Marginalized People in the United States and Europe, 1990–2014. Vector-Borne Zoonotic Dis. 2016;16(7):435–444. doi: 10.1089/vbz.2015.1863 27159039 10.1089/vbz.2015.1863

[7] Lau CL, Watson CH, Lowry JH, David MC, Craig SB, Wynwood SJ, et al Human Leptospirosis Infection in Fiji: An Eco-epidemiological Approach to Identifying Risk Factors and Environmental Drivers for Transmission. PLoS Negl Trop Dis. 2016;10(1):e0004405 doi: 10.1371/journal.pntd.0004405 26820752 10.1371/journal.pntd.0004405 PMC4731082

[8] MSAL. Enfermedades Infecciosas, Leptospirosis. Guia para el Equipo de Salud. Cdad. Autónoma de Bs. As., Argentina: Dirección de Epidemiología—Ministerio de Salud de la Nación; 2014. Available from: https://www.argentina.gob.ar/salud.

[9] Navegantes de Araújo W, Finkmoore B, Ribeiro GS, Reis RB, Felzemburgh RDM, Hagan JE, et al Knowledge, attitudes, and practices related to leptospirosis among urban slum residents in Brazil. Am J Trop Med Hyg. 2013;88(2):359–363. doi: 10.4269/ajtmh.2012.12-0245 23269657 10.4269/ajtmh.2012.12-0245 PMC3583330

[10] Maciel EAP, de Carvalho ALF, Nascimento SF, de Matos RB, Gouveia EL, Reis MG, et al Household transmission of Leptospira infection in urban slum communities. PLoS Negl Trop Dis. 2008;2(1):e154 doi: 10.1371/journal.pntd.0000154 18357340 10.1371/journal.pntd.0000154 PMC2270796

[11] Reis RB, Ribeiro GS, Felzemburgh RDM, Santana FS, Mohr S, Melendez AXTO, et al Impact of environment and social gradient on Leptospira infection in urban slums. PLoS Negl Trop Dis. 2008;2(4):e228 doi: 10.1371/journal.pntd.0000228 18431445 10.1371/journal.pntd.0000228 PMC2292260

[12] Sarkar U, Nascimento S, Barbosa R, For MbCCIoRF, Leptospirosis During an Urban Epidemicins R, Nuevo H, et al Population-based case-control investigation of risk factors for leptospirosis during an urban epidemic. Am J Trop Med Hyg. 2002;66(5):605–10. doi: 10.4269/ajtmh.2002.66.605 12201599 10.4269/ajtmh.2002.66.605

[13] Costa F, Hagan JE, Calcagno J, Kane M, Torgerson P, Martinez-Silveira MS, et al Global Morbidity and Mortality of Leptospirosis: A Systematic Review. PLoS Negl Trop Dis. 2015;9(9):0–1. doi: 10.1371/journal.pntd.0003898 10.1371/journal.pntd.0003898 PMC4574773 26379143

[14] Torgerson PR, Hagan JE, Costa F, Calcagno J, Kane M, Martinez-Silveira MS, et al Global Burden of Leptospirosis: Estimated in Terms of Disability Adjusted Life Years. PLoS Negl Trop Dis. 2015;9(10):e0004122 doi: 10.1371/journal.pntd.0004122 26431366 10.1371/journal.pntd.0004122 PMC4591975

[15] Lau CL, Smythe LD, Craig SB, Weinstein P. Climate change, flooding, urbanisation and leptospirosis: Fuelling the fire? Trans R Soc Trop Med Hyg. 2010;104(10):631–638. doi: 10.1016/j.trstmh.2010.07.002 20813388 10.1016/j.trstmh.2010.07.002

[16] Schneider MC, Leonel DG, Hamrick PN, Pacheco De Caldas E, Velásquez RT, Mendigaña Paez FA, et al Leptospirosis in Latin America: exploring the first set of regional data. Rev Panam Salud Publica. 2017;41:1–9. 10.26633/RPSP.2017.81 PMC6645204 31384245

[17] Costa F, Martinez-Silveira MS, Hagan JE, Hartskeerl RA, Dos Reis MG, Ko AI. Surveillance for Leptospirosis in the Americas, 1996-2005: A Review of Data from Ministries of Health. Rev Panam Salud Pública. 2012;32(3):169–77. doi: 10.1590/S1020-49892012000900001 23183556 10.1590/s1020-49892012000900001 PMC3970205

[18] MSAL. Boletín Integrado de Vigilancia N° 393—SE 1—Enero de 2018. Secretaría de Promoción y programas sanitarios—Ministerio de Salud de la Nación; 2018. Available from: https://www.argentina.gob.ar/salud.

[19] MSAL. Boletín Integrado de Vigilancia N° 343—SE 2—Enero de 2017. Secretaría de Promoción y programas sanitarios—Ministerio de Salud de la Nación; 2017. Available from: https://www.argentina.gob.ar/salud.

[20] MSAL. Boletín Integrado de Vigilancia N° 293—SE 2—Enero de 2016. Secretaría de Promoción y programas sanitarios—Ministerio de Salud de la Nación; 2016. Available from: https://www.argentina.gob.ar/salud.

[21] MSAL. Boletín Integrado de Vigilancia N° 241—SE 1—Enero de 2015. Secretaría de Promoción y programas sanitarios—Ministerio de Salud de la Nación; 2015. Available from: https://www.argentina.gob.ar/salud.

[22] MSAL. Boletín Integrado de Vigilancia N° 200—SE 52—Diciembre de 2013. Secretaría de Promoción y programas sanitarios—Ministerio de Salud de la Nación; 2013. Available from: https://www.argentina.gob.ar/salud.

[23] MSAL. Boletín Integrado de Vigilancia N° 119—SE 19—Mayo de 2012. Secretaría de Promoción y programas sanitarios—Ministerio de Salud de la Nación; 2012. Available from: https://www.argentina.gob.ar/salud.

[24] Vanasco NB, Schmeling MF, Lottersberger J, Costa F, Ko AI, Tarabla HD. Clinical characteristics and risk factors of human leptospirosis in Argentina (1999-2005). Acta Trop. 2008;107(3):255–258. doi: 10.1016/j.actatropica.2008.06.007 18671932 10.1016/j.actatropica.2008.06.007

[25] Vanasco NB, Sequeira G, Dalla Fontana ML, Fusco S, Sequeira MD, Enria D. Report on a leptospirosis outbreak in the city of Santa Fe, Argentina, March-April 1998. Rev Panam Salud Pública. 2000;7(1):35–40. 10715972 10.1590/s1020-49892000000100006

[26] CCLA-AAVLD. Informe sobre Leptospirosis en la República Argentina. Buenos Aires: Comisión Científica sobre Leptospirosis en la República Argentina (CCLA)—Asociación Argentina de Veterinarios de Laboratorio de Diagnóstico (AAVLD). Fundación Mundo Sano; 2002. Publicación monográfica: 3.

[27] Cacchione RA, Cascelli ES, Martínez ES. Encuesta serólogica sobre leptospirosis humana en Argentina [Serologic survey on human leptospirosis in Argentina]. Rev Asoc Argent Microbiol. 1975;7(1):21–27. 1208897

[28] Cudós MC, Landolt N, Jacob P, Schmeling MF, Chiani Y, Brazza S, et al Vigilancia Intensificada De Leptospirosis En Santa Fe Y Entre Ríos (2012-2013). Rev Argent Salud Pública. 2014;5(18):24–30.

[29] Cudós MC, Schmeling MF, Chiani Y, Landolt N, Jacob P, Grubert S, et al Descripción de tres brotes de leptospirosis en escenarios epidemiológicos emergentes In: 1er Simp. Nac. Vigil. la Salud. Buenos Aires; 2013.

[30] Vanasco NB, Fusco S, Zanuttini JC, Manattini S, Dalla Fontana ML, Prez J, et al [Outbreak of human leptospirosis after a flood in Reconquista, Santa Fe, 1998]. Rev Argent Microbiol. 2002;34(3):124–131. 12415894

[31] Lugova H, Wallis S. Cross-Sectional Survey on the Dengue Knowledge, Attitudes and Preventive Practices Among Students and Staff of a Public University in Malaysia. J Community Health. 2017;42(2):413–420. doi: 10.1007/s10900-016-0270-y 27696137 10.1007/s10900-016-0270-y

[32] Arbiol J, Orencio P, Romena N, Nomura H, Takahashi Y, Yabe M. Knowledge, Attitude and Practices towards Leptospirosis among Lakeshore Communities of Calamba and Los Baños, Laguna, Philippines. Agriculture. 2016;6 (2)(18):12 pp. doi: 10.3390/agriculture6020018

[33] Sakinah SNS, Suhailah S, Jamaluddin TZMT, Norbaya SM, Malina O. Seroprevalence of Leptospiral Antibodies and Knowledge, Attitude and Practices of Leptospirosis To Non High Risk Group in Selangor. Int J Public Heal Clin Sci. 2015;2(1):92–104.

[34] Samarakoon YM, Gunawardena N. Knowledge and self-reported practices regarding leptospirosis among adolescent school children in a highly endemic rural area in Sri Lanka. Rural Remote Health. 2013;13(4):12 pp. 24144327

[35] Mohan ARM, Chadee DD. Knowledge, attitudes and practices of Trinidadian households regarding leptospirosis and related matters. Int Health. 2011;3(2):131–137. doi: 10.1016/j.inhe.2011.03.002 24038186 10.1016/j.inhe.2011.03.002

[36] Essi MJ, Njoya O. Point de vue L’Enquête CAP (Connaissances, Attitudes, Pratiques) en Recherche Médicale. Heal Sci Dis Vol. 2013;14(2):3 pp.

[37] Rahim MS, Nazri MS, Rusli MA. Town Service Workers’ Knowledge, Attitude and Practice towards Leptospirosis. Brunei Darussalam J Heal. 2012;5:1–12.

[38] IPEC-INDEC. Censo Nacional de Población, Hogares y Viviendas 2010; Provincia de Santa Fe;. Available from: https://www.santafe.gov.ar/index.php/web/content/view/full/163622/(subtema)/93664.

[39] QGIS Development Team. QGIS Geographic Information System 3.0 Girona; 2018. Available from: http://qgis.osgeo.org.

[40] Congedo L. Semi-Automatic Classification plugin for QGIS Sapienza Univ. 2013;Rome:1–25.

[41] IPEC-INDEC. Censo Nacional de Población, Hogares y Viviendas 2010; Ciudad de Santa Fe por Radio Censal;. Available from: https://www.santafe.gov.ar/index.php/web/content/view/full/163619/(subtema)/93664.

[42] Rittaca M, Víttori M. La Vuelta del Paraguayo, el más islero de la ciudad; 2013. Available from: http://www.ellitoral.com/index.php/id{_}um/85169-la-vuelta-del-paraguayo-el-ms-islero-de-la-ciudad.

[43] Rittaca M, Vittori M, Vittori MS. El agua, el mayor de los problemas; 2014. Available from: http://www.ellitoral.com/index.php/id{_}um/105176-el-agua-el-mayor-de-los-problemas.

[44] Agampodi SB, Agampodi TC, Thalagala E, Perera S, Chandraratne S, Fernando S. Do People Know Adequately about Leptospirosis? A Knowledge Assessment Survey in Post outbreak Situation in Sri Lanka. Int J Prev Med. 2015;1(3):158–163. PMC3075525 21566785

[45] Davlin SL, Lapiz SM, Miranda ME, Murray KO. Knowledge, attitudes, and practices regarding rabies in Filipinos following implementation of the Bohol Rabies Prevention and Elimination Programme. Epidemiol Infect. 2014;142(07):1476–1485. doi: 10.1017/S0950268813002513 24093635 10.1017/S0950268813002513 PMC9151205

[46] R Core Team. R: A Language and Environment for Statistical Computing; 2017. Available from: http://www.r-project.org/.

[47] Bates D, Mächler M, Bolker B, Walker S. Fitting Linear Mixed-Effects Models using lme4. JSS J Stat Softw. 2014;67(1):48.

[48] Halekoh U, Højsgaard S. A Kenward-Roger Approximation and Parametric Bootstrap Methods for Tests in Linear Mixed Models—The R Package pbkrtest. J Stat Softw. 2014;59(9):1–32. doi: 10.18637/jss.v059.i0926917999

[49] Burnham KP, Anderson DR, Huyvaert KP. AIC model selection and multimodel inference in behavioral ecology: Some background, observations, and comparisons. Behav Ecol Sociobiol. 2011;65(1):23–35. doi: 10.1007/s00265-010-1029-6

[50] Barton K. MuMIn: Multi-model inference. R package version 1.15.6.; 2016. Available from: http://cran.r-project.org/package=MuMIn.

[51] Mason MR, Gonzalez M, Hodges JS, Muñoz-Zanzi C. Protective practices against zoonotic infections among rural and slum communities from South Central Chile. BMC Public Health. 2015;15(1):1–15. 26215091 10.1186/s12889-015-1964-2 PMC4517625

[52] Abiayi EA, Inabo HI, Jatau ED, Makinde AA, Sar TT, Ugbe DA, et al Knowledge, attitudes, risk factors and practices (KARP) that favor Leptospira infection among abattoir workers in North Central Nigeria. Asian J Epidemiol. 2015;8(4):104–113. doi: 10.3923/aje.2015.104.113

[53] Chiani Y, Jacob P, Varni V, Landolt N, Schmeling MF, Pujato N, et al Isolation and clinical sample typing of human leptospirosis cases in Argentina. Infect Genet Evol. 2016;37:245–251. doi: 10.1016/j.meegid.2015.11.033 26658064 10.1016/j.meegid.2015.11.033

[54] Lelu M, Muñoz-Zanzi C, Higgins B, Galloway R. Seroepidemiology of leptospirosis in dogs from rural and slum communities of Los Rios Region, Chile. BMC Vet Res. 2015;11(1). doi: 10.1186/s12917-015-0341-9 25880871 10.1186/s12917-015-0341-9 PMC4329218

[55] Chiani Y. Desarrollo y Validación de Técnicas Diagnósticas de Leptospirosis Canina [M.Sc. Thesis]. Universidad Nacional del Litoral; 2013. Available from: http://bibliotecavirtual.unl.edu.ar:8080/tesis/handle/11185/434.

[56] MSAL. Boletín Integrado de Vigilancia N° 311—SE 21—Mayo de 2016. Secretaría de Promoción y programas sanitarios—Ministerio de Salud de la Nación; 2016. Available from: https://www.argentina.gob.ar/salud.

[57] MSAL. Boletín Integrado de Vigilancia N° 315—SE 25—Junio de 2016. Secretaría de Promoción y programas sanitarios—Ministerio de Salud de la Nación; 2016. Available from: https://www.argentina.gob.ar/salud.

[58] INER. Vigilancia de leptospirosis por el laboratorio en argentina (2016). Santa Fe: Laboratorio de Referencia Nacional de Leptospirosis del INER “Dr. E. Coni” Coordinador de la Red Nacional de Laboratorios de Leptospirosis; 2016. Available from: http://www.anlis.gov.ar/iner/?page{_}id=1665.

[59] Libraty DH, Myint KSA, Murray CK, Gibbons RV, Mammen MP, Endy TP, et al A comparative study of leptospirosis and dengue in Thai children. PLoS Negl Trop Dis. 2007;1(3):e111 doi: 10.1371/journal.pntd.0000111 18160980 10.1371/journal.pntd.0000111 PMC2154391

[60] LaRocque RC, Breiman RF, Ari MD, Morey RE, Janan FA, Hayes JM, et al Leptospirosis during dengue outbreak, Bangladesh. Emerg Infect Dis. 2005;11(5):766–769. doi: 10.3201/eid1105.041212 15890136 10.3201/eid1105.041212 PMC3320387

[61] Sanders EJ, Rigau-Pérez JG, Smits HL, Deseda CC, Vorndam VA, Aye T, et al Increase of leptospirosis in dengue-negative patients after a Hurricane in Puerto Rico in 1966. Am J Trop Med Hyg. 1999;61(3):399–404. doi: 10.4269/ajtmh.1999.61.399 10497979 10.4269/ajtmh.1999.61.399

[62] Heshmat R, Abdollahi Z, Ghotbabadi FS, Rostami M, Shafiee G, Qorbani M, et al Nutritional knowledge, attitude and practice toward micronutrients among Iranian households: the NUTRI-KAP survey. J Diabetes Metab Disord. 2015;15(1):42 doi: 10.1186/s40200-016-0260-8 27709106 10.1186/s40200-016-0260-8 PMC5050604

[63] Teferi J, Shewangizaw Z. Assessment of knowledge, attitude, and practice related to epilepsy: A community-based study. Neuropsychiatr Dis Treat. 2015;11:1239–1246. doi: 10.2147/NDT.S82328 26056455 10.2147/NDT.S82328 PMC4446018

[64] Barkat A, Helali J, Rahman M, Majid M, Bose M. Knowledge attitude perception and practices relevant to the utilization of emergency obstetric care services in Bangladesh: a formative study.; 1995.

[65] Quina CR, Almazan JU, Tagarino JB. Knowledge, Attitudes, and Practices of Leptospirosis in Catbalogan City, Samar, Philippines. Am J Public Heal Res. 2014;2(3):91–98. doi: 10.12691/ajphr-2-3-5

[66] Grier S, Bryant CA. Social Marketing in Public Health. Annu Rev Public Health. 2005;26(1):319–339. doi: 10.1146/annurev.publhealth.26.021304.144610 15760292 10.1146/annurev.publhealth.26.021304.144610

[67] Beat Chagas;. Available from: http://beatchagas.org/.

[68] Sanmartino M, Amieva C, Balsalobre A, Carrillo A, Marti G, Medone P, et al. Hablamos de Chagas: aportes para re-pensar la problemática con una mirada integral. Ciudad Autónoma de Buenos Aires: CONICET—Consejo Nacional de Investigaciones Científicas y Técnicas; 2015.

[69] Parkes MW, Bienen L, Breilh J, Hsu LN, McDonald M, Patz JA, et al All Hands on Deck: Transdisciplinary Approaches to Emerging Infectious Disease. Ecohealth. 2005;2(4):258–272.

[70] Bardosh K. Global aspirations, local realities: The role of social science research in controlling neglected tropical diseases. Infect Dis Poverty. 2014;3(1):1–15. 25320672 10.1186/2049-9957-3-35 PMC4197218

