# Supplementary material for: Knowledge, attitudes and practices (KAP) regarding leptospirosis among residents of riverside settlements of Santa Fe, Argentina
Source: PLoS Negl Trop Dis. 2018 May 7;12(5):e0006470. doi: 10.1371/journal.pntd.0006470 (PMC5957447; doi:10.1371/journal.pntd.0006470)
Supplement: S1 Table — The questions used in the construction of knowledge score are highlighted in yellow, those used in the construction of attitudes score are highlighted in green, and those used in the practices score are highlighted in pink. (PDF) [file pntd.0006470.s001.pdf]

ENCUESTA INDIVIDUAL

**Nombre encuestador:**	**Fecha:**	**Nro. Encuesta:**	**Lugar:**	**Evacuado**	Sí	No

1. **PARA EMPEZAR NECESITO UNOS DATOS SOBRE UD:**

**Encuestado**	**Edad**	**Sexo**		**Estudios**				**Ocupación**
		M	F	PI<br>PC	SI<br>SC	TI<br>TC	UI<br>UC	

Nota: En la primera columna se coloca la situación del encuestado respecto al jefe de hogar, si es cónyuge, hijo, tío, primo, amigo, etc.

2. **¿REALIZA USTED ALGUNA DE ESTAS ACTIVIDADES?**

**Actividad**	**¿Qué tan seguido?**			**Comentarios**
	**Frecuentemente**	**Raramente**	**Nunca**	
Pescar				
Recolectar leña				
Cazar				
Desmalezar				
Otra/s				

Frecuentemente = 0; Raramente = 1; Nunca = 2

3. **¿QUÉ TIPO DE CALZADO USA CUANDO LAS REALIZA?**

Descalzo		Zapatillas/Otro		Botas	

Descalzo = -2; Zapatillas = -1; Botas = 0

4. **¿VA A LA ISLA?** (No: Pase a 6)

	Sí		No

5. **¿PASA LA NOCHE EN LA ISLA?**

	Sí		No

6. **SI HA LLOVIDO MUCHO Y UD. NECESITA SALIR DE LA CASA, ¿LE TOCA ATRAVESAR CHARCOS GRANDES PARA LLEGAR A DESTINO?**

	Sí		No

7. **¿EN ESTOS CASOS SE MOJA LOS PIES?**

	Sí		No

8. **ESTO LE SUCEDE. . .**

Siempre	Casi siempre	A veces	Casi Nunca	Nunca

9. **¿USA USTED AGUA DEL RÍO/CANAL/LAGUNA/CAVA PARA REGAR/LAVAR/DARLE DE BEBER A LOS ANIMALES?** (No: Pase a 11)

	Sí		No

10. **¿DE QUÉ LUGAR LA SACA?**

11. **¿NADA O SE REFRESCA EN EL RÍO, LAGUNA U OTRO LUGAR DE LA ZONA?** (No: Pase a 13)

	Sí		No

12. **¿EN QUÉ LUGAR? (nombre y ubicación del lugar)**

13. **¿ALGUNA VEZ ESCUCHÓ HABLAR DE LEPTOSPIROSIS?**
(No: Pase a 30)

	Sí		No

14. **PARA UD ¿QUÉ ES LA LEPTOSPIROSIS?**
No deben leerse las opciones (Máx = 3 puntos)

	No sé		Los perros se enferman
	Una enfermedad		Las personas sangran
	Una enfermedad transmitida por las ratas		Viene de las alcantarillas
	Una enfermedad que mata		Mucha gente se contagia
	Una enfermedad de trabajo		Transmitida por mosquitos
	Una enfermedad que da fiebre		Es infecciosa
	La persona se pone amarilla		Causada por animales
	Asociada a lluvias/Inundaciones		Otra/s

Cual/es?:

15. **¿DÓNDE HA OÍDO HABLAR DE LEPTOSPIROSIS?**
No deben leerse las opciones.

	Centro de salud, dispensario, hospital		Trabajo
	Medios (televisión, radio, diarios, etc)		Escuela
	Conocido, familiar		Vecinal
	Otro/s		

16. **¿CUANTAS PERSONAS EN LA CIUDAD DE SANTA FE Y ALREDEDORES CREE USTED QUE SE ENFERMAN AL AÑO?**

	Muchas		Algunas		Pocas		No sé

17. **¿CREE UD. QUE EN ALGÚN MOMENTO UNA GRAN CANTIDAD DE PERSONAS PODRÍAN ENFERMARSE DE LEPTOSPIROSIS?**
(Cree que pueda haber un brote de Leptospirosis)

	Sí		Tal vez		No		No sé

18. **¿CONOCE A ALGUIEN QUE HAYA TENIDO ESTA ENFERMEDAD?** (No: pase a 23)

	Sí		No

19. **¿CUÁNTAS PERSONAS?**

20. **ALGUIEN DE SU BARRIO?**

	Sí		No

¿Cuantos?:

21. **¿ALGUIEN DE SU CASA?**

	Sí		No

¿Cuantos?:

22. **¿ALGUNA DE ELLAS MURIÓ POR ESTA ENFERMEDAD?**
(Sí: Pase a 25)

	Sí		No

23. **¿SE PUEDEN CURAR LAS PERSONAS DE ESTA ENFERMEDAD?**

	Sí		No		No sé

24. **¿PUEDE UNA PERSONA MORIRSE POR ESTA ENFERMEDAD?**

	Sí		No		No sé

25. **¿QUÉ SIENTE UNA PERSONA CUANDO TIENE ESTA ENFERMEDAD?** (¿Cuáles son los síntomas?)
(No deben leerse las opciones / Máx = 5 puntos)

	No sé		Dolor cabeza
	Fiebre		Problema renal
	Dolor cuerpo		Dolor piernas
	Malestar		Otro/s

¿Cuál/es?:

26. **¿CÓMO LE PARECE QUE UNA PERSONA PUEDE AGARRARSE ESTA ENFERMEDAD?** (¿Cómo se contagia?)
(No deben leerse las opciones / Máx = 4 puntos)

	No sé		Orina ratas
	Contacto con animales enfermos		Contacto con agua estancada
	Limpiando zanjas		Al desmalezar
	Picadura mosquitos		Contacto con basura
	Por comida/agua contaminada		Por heridas
	Por andar descalzo		Por ir a la isla
	Otro/s:		

27. **¿QUÉ ANIMALES CREE USTED PUEDEN TRANSMITIR ESTA ENFERMEDAD A LAS PERSONAS?**
(Marcar los mencionados / Máx = 4 puntos)

	No sé		Gallinas, aves
	Perros		Ratas, lauchas, ratones
	Gatos		Caballos
	Vacas		Cabras, ovejas
	Cerdos		Otro/s

28. **¿QUÉ TIENEN QUE HACER LAS PERSONAS PARA NO CONTAGIARSE LEPTOSPIROSIS?** (¿Cómo pueden prevenirla?)
(No deben leerse las opciones / Máx = 4 puntos)

	No sé
	Matar/Controlar ratas
	Limpiar zanjas protegiéndose
	Vacunándose (quimioprofilaxis)
	Evitar contacto con animales enfermos
	Evitar contacto con agua estancada
	Evitar agua/comida contaminada
	Utilizar botas/guantes
	Evitar contacto con basura
	Otro/s

¿Cual/es?:

29. **SI AUMENTA EL NÚMERO DE CASOS DE LEPTOSPIROSIS**

**¿Ud. Podría tomar las medidas necesarias para no contagiarse?***	**Sí**	**No sé**	**No**	¿Porqué?
**¿Tendría miedo de contagiarse?***	**Sí**	**No sé**	**No**	¿Porqué?
¿Confiaría en lo que dicen los medios sobre cuán grave es la situación?	Sí	No sé	No	¿Porqué?
¿Piensa que los gobiernos llegarían pronto a brindar tratamiento a las personas?	Sí	No sé	No	¿Porqué?

* Utilizadas en la construcción del score de actitudes.

30. **¿HA OIDO HABLAR DEL DENGUE?** (No: Pase a 34)

	Sí		No

31. **AL COMPARAR EL DENGUE CON LA LEPTOSPIROSIS...**

	**Leptospirosis**	**Dengue**	**Igual**	**No sé**
¿Cuál le parece que es una enfermedad más grave?				
¿Cuál piensa que Ud. tiene más riesgo de contraer?				
¿Cuál le parece que afecta a una mayor cantidad de personas?				

32. **¿UD SUELE IR AL MÉDICO?**

	Sí		No

33. **SI TUVIERA ALGUNOS DE ESTOS SÍNTOMAS ¿UD IRÍA AL MÉDICO?** (Leer las opciones)

**Síntoma**	**Sí**	**No**	**Tal vez**
Dolor de cabeza			
Nauseas y vómitos			
**Fiebre***			
**Fiebre alta (si respondió No en la anterior)***			
Malestar general (sentirse mal)			
Dificultad para respirar			
Dolor en el cuerpo (articulaciones/pantorrillas)			
Enrojecimiento de los ojos			
Sarpullido o ronchas en el cuerpo			
**Si tiene 2 o más síntomas al mismo tiempo****			

* Utilizadas en la construcción del score de actitudes.
** Preguntar si respondió **No** en todos los casos anteriores.

34. **CUANDO NECESITA ATENCIÓN MÉDICA ¿A QUÉ LUGAR VA?** (No deben leerse las opciones.)

	Dispensario	¿Cuál?:
	Centro de salud	¿Cuál?:
	Hospital	¿Cuál?:
	Sanatorio/Clínica<br>Otro/s	¿Cuál?:<br>¿Cuál?:

35. **TIENE UD. ALGUNA QUEJA SOBRE EL SISTEMA DE SALUD? ¿VE ALGÚN PROBLEMA PARA RECIBIR LA ATENCIÓN MÉDICA NECESARIA?**

	Sí		No

¿Cuál?:

36. **COMENTARIOS U OBSERVACIONES EN GENERAL**
