# Supplementary material for: Knowledge, attitudes and practices (KAP) regarding leptospirosis among residents of riverside settlements of Santa Fe, Argentina
Source: PLoS Negl Trop Dis. 2018 May 7;12(5):e0006470. doi: 10.1371/journal.pntd.0006470 (PMC5957447; doi:10.1371/journal.pntd.0006470)
Supplement: S2 Table — (PDF) [file pntd.0006470.s002.pdf]

S2_Table. Frequencies (%) of risk practices and situations avoided by male and female respondents (n=113).

Variable	Female n=69	Male n=44	P
**Fishing**			<0.001
Frequently	18 (26.1)	25 (56.8)	
Rarely	7 (10.1)	9 (20.5)	
Never	44 (63.8)	10 (22.7)	
**Hunting**			<0.001
Frequently	3 (4.3)	11 (25.0)	
Rarely	1 (1.4)	4 (9.1)	
Never	65 (94.2)	29 (65.9)	
**Gardening**			0.42
Frequently	27 (39.1)	20 (45.5)	
Rarely	2 (2.9)	3 (6.8)	
Never	40 (58.0)	21 (47.7)	
**Collecting firewood**			0.68
Frequently	23 (33.3)	18 (40.9)	
Rarely	6 (8.7)	4 (9.1)	
Never	40 (58.0)	22 (50.0)	
**Going to the river islands**			0.001
Yes	25 (36.2)	30 (68.2)	
No	44 (63.8)	14 (31.8)	
**Spent the night at the island**			0.001
Yes	16 (23.2)	24 (54.5)	
No	53 (76.8)	20 (45.5)	
**Walk through flood water**			0.18
Yes	50 (72.5)	37 (84.1)	
No	19 (27.5)	7 (15.9)	
**Getting feet wet on flood water**			1.00
Yes	41 (59.4)	27 (61.4)	
No	28 (40.6)	17 (38.6)	
**Use water of the river to clean/drink**			0.68
Yes	20 (29.0)	15 (34.1)	
No	49 (71.0)	29 (65.9)	
**Swim in the river/flood water**			<0.001
Yes	19 (27.5)	28 (63.6)	
No	50 (72.5)	16 (36.4)	
