# Supplementary material for: Knowledge, attitudes and practices (KAP) regarding leptospirosis among residents of riverside settlements of Santa Fe, Argentina
Source: PLoS Negl Trop Dis. 2018 May 7;12(5):e0006470. doi: 10.1371/journal.pntd.0006470 (PMC5957447; doi:10.1371/journal.pntd.0006470)
Supplement: S1 Fig — (PDF) [file pntd.0006470.s003.pdf]

"Cuidar nuestro ambiente, es cuidar nuestra salud"

LEPTOSPIROSIS

Una enfermedad producida por una bacteria llamada Leptospira que puede estar presente en la orina de ciertos animales: perros, gatos, vacas, cerdos, caballos, ovejas, ratas, lauchas,nutrias, comadrejas, carpinchos, entre otros.

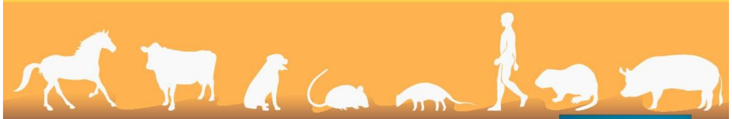

UNIVERSIDAD NACIONAL DEL LITORAL
Secretaría de Extensión

LEPTOSPIROSIS

¿Cómo se contagia?

· Por contacto directo con orina, líquidos y tejidos de animales infectados.
· Por contacto con agua, barro, o ambientes contaminados con la orina de dichos animales.

¿Cómo podemos cuidarnos?

· Evitando que los niños jueguen en el barro, charcos, cunetas y basurales.
· Usando siempre calzado (preferentemente botas de goma) al andar por lugares inundados.
· Combatiendo ratas y lauchas en las viviendas.
· Instalando potreros y gallineros lejos de la vivienda.
· Utilizando guantes y botas de goma al realizar actividades donde nos podemos contagiar, tales como: pesca, ordeñe, cría o faena de animales, limpieza de zanjas y desmalezado de terrenos, siembra y cosecha, cirujeo, etc.

¿Qué hacer si nos enfermamos?

· Ir al médico cuando tenemos síntomas parecidos a los de una gripe (fiebre, dolor de cabeza, dolores musculares y malestar general).
· Contarle al médico si hemos realizado alguna de las actividades mencionadas.

Proyecto de Extensión de Interés Social – Facultad de Humanidades y Ciencias.
"Socioecología de la enfermedad de Leptospirosis en comunidades costeras de Santa Fe"
